# Good results with the Ponseti method: A multicenter study of 162 clubfeet followed for 2–5 years

**DOI:** 10.3109/17453674.2012.693015

**Published:** 2012-06-04

**Authors:** Christian Sætersdal, Jonas M Fevang, Lars Fosse, Lars B Engesæter

**Affiliations:** ^1^Department of Orthopedic Surgery, Haukeland University Hospital, Bergen, Norway; ^2^Department of Pediatric Orthopedics, Astrid Lindgren Children’s Hospital, Karolinska University Hospital, Stockholm, Sweden; ^3^Institute of Surgical Sciences, University of Bergen, Bergen, Norway; Correspondence: christian.saetersdal@helse-bergen.no

## Abstract

**Background and purpose:**

In 2002–2003, several hospitals in Norway introduced the Ponseti method for treating clubfoot. The present multicenter study was conducted to evaluate the initial results of this method, and to compare them to the good results reported in the literature.

**Patients and methods:**

116 children with 162 congenital idiopathic clubfeet who were born between 2004 and 2006 were treated with the Ponseti method at 8 hospitals in Norway. All children were prospectively registered at birth, and 116 feet were assessed according to Pirani before treatment was started. 63% used a standard bilateral foot abduction brace, and 32% used a unilateral above-the-knee brace. One of the authors examined all feet at a mean age of 4 years. At follow-up, all feet were assessed by Pirani’s scoring system, and range of motion of the foot and ankle was measured.

**Results:**

At follow-up, 77% of the feet had a Pirani score of 0.5 or better, good dorsiflexion and external rotation, and no forefoot adduction. An Achilles tenotomy had been performed in 79% of the feet. Compliance to any brace was good; only 7% were defined as non-compliant. Extensive soft tissue release had been performed in 3% of the feet.

We found no statistically significant differences between the two braces, except a tendency of better Pirani score in the group using the bilateral foot abduction brace, and a tendency of better compliance in patients using the unilateral brace. Better Pirani scores were found in children who were treated at the largest hospitals.

**Interpretation:**

After introducing the Ponseti method in Norway, the clinical outcome was good and in accordance with the reports from single centers. Only 5 feet needed extensive surgery during the first 4 years of life.

The methods of treating clubfoot have varied over the years and between the different hospitals in Norway. The results reported have not been satisfactory, as 75% of the feet needed posterior or postero-medial release (Nesse et al. 1996). Thus, orthopedic surgeons treating clubfoot in Norway decided to start with the Ponseti method, which has shown promising short-term and long-term results (Laaveg and Ponseti 1980, Cooper and Dietz 1995, Herzenberg et al. 2002). The Ponseti method of treating clubfoot was introduced at several hospitals in Norway in 2002 and 2003.

A foot abduction brace is a crucial part of the Ponseti treatment, and it is well documented that the brace prevents a clubfoot from relapsing (Dobbs et al. 2004, Morcuende et al. 2004). The brace recommended by Ponseti is a bilateral foot abduction brace. Many hospitals in Norway have traditionally used a custom-made unilateral above-the-knee dynamic brace to prevent relapse. Some of these hospitals continued to offer this brace to children with clubfoot, even after the introduction of the Ponseti casting method.

Norway is a small country regarding population (4.9 million inhabitants), but it has a relatively large area and none of the hospitals were responsible for treating more than 10 newborns with clubfoot every year in this study.

We evaluated our results and compared them to the good short-term and long-term results reported in the literature. We also compared the unilateral above-the-knee brace with the standard bilateral foot abduction brace regarding both clinical outcome and compliance to brace use. Finally, we determined whether the results were influenced by the number of clubfeet treated at each hospital.

## Patients and methods

In this multicenter clinical study, all 134 newborns with idiopathic congenital clubfoot treated at 8 hospitals in Norway during the period 2004–2006 met the inclusion criteria. All the children were registered prospectively from birth, and 116 feet were assessed according to Pirani before treatment was started. In March 2009, one of the authors (CS) visited all 8 hospitals and performed a standardized clinical examination of the patients.

### Patients

**Figure 1.**
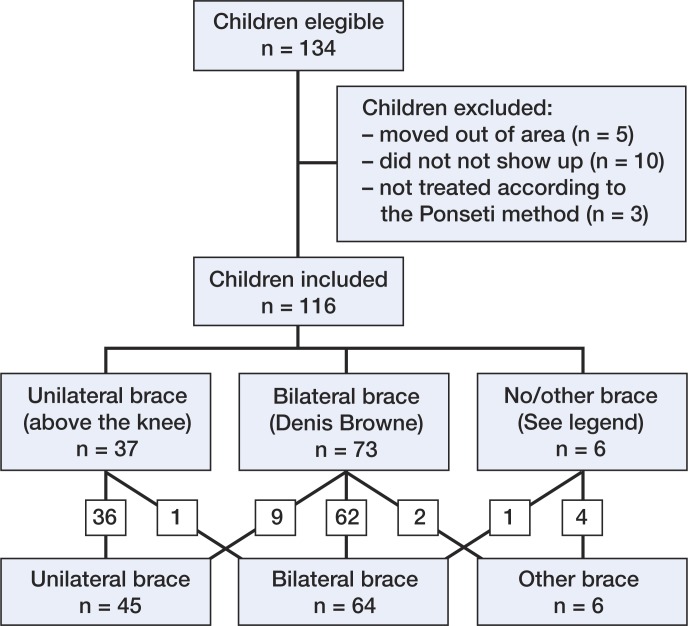
Overview of children enrolled in the study, and type of brace used. No/other brace: 1 child did not use any brace; 5 children used a below-the-knee unilateral brace of different types; 1 changed to a bilateral brace. Bilateral brace: 9 changed to a unilateral brace and 2 to another type of brace. Unilateral brace: 1 changed to a bilateral brace.

Of the 134 children, 15 had either moved out of the area or failed to show up, and 3 children had initial treatment that differed too much from Ponseti’s descriptions. Accordingly, 116 individuals (72% boys) were included in this study (Figure 1). 46 children had bilateral disorder and 70 had unilateral disorder; thus, the total number of clubfeet examined was 162. Each hospital enrolled between 7 and 25 children in the study.

### Cast

78% of the feet were treated with a semi-rigid fibreglass cast, and the rest with plaster of Paris. On average, the first cast was applied on the second day of life (range 0–9 days). 4 children with unilateral clubfoot had a delayed diagnosis, and the first cast was applied on days 18, 37, 58, and 60. These 4 children had mild clubfoot deformity with low Pirani score and needed only 3–5 casts to correct the deformity.

### Brace

**Figure 2.**
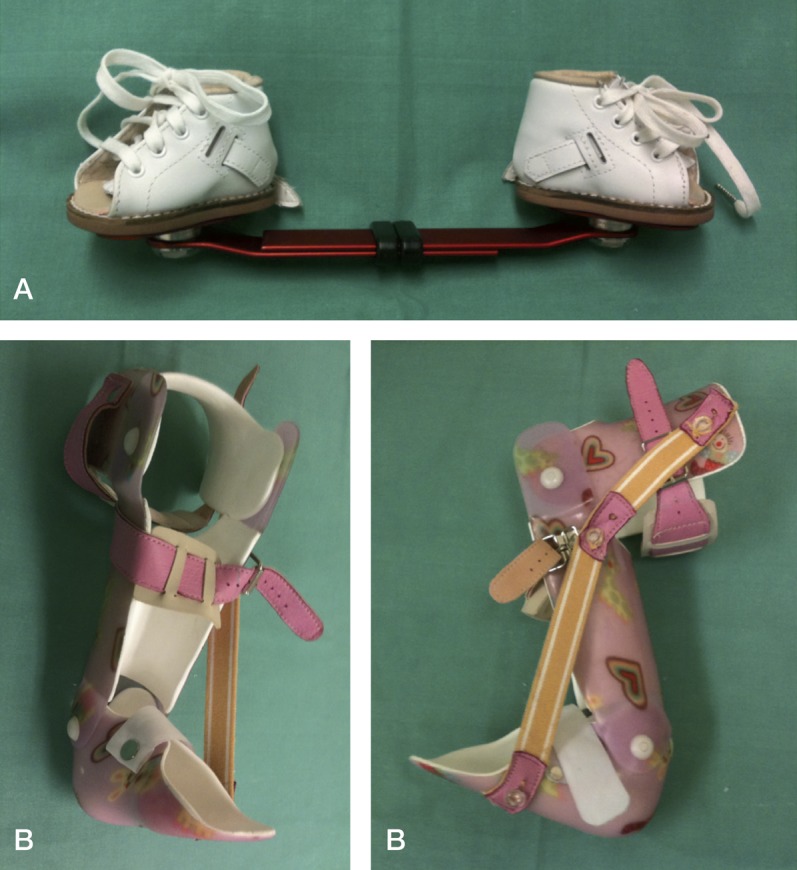
A. Bilateral foot abduction brace. B. Unilateral foot abduction brace, side and frontal view.

62% of the children used a standard, bilateral foot abduction brace (FAB) as recommended by Ponseti, immediately after the casting period (Figure 2A). In 55% of the children using the FAB, no changes in brace use were made, while 30% changed to another bilateral FAB from a different producer (for example, from Markell to Mitchell). 12% of the children with bilateral FAB changed to a different type of brace—the flexible custom-made unilateral above-the-knee brace, which has traditionally been used at some hospitals in Norway (Figure 2b). 3% of the children with bilateral FAB changed to a softcast/scotchcast removable brace/cast.

32% of the children were at first fitted into the unilateral above-the-knee brace. 1 of these children changed to a bilateral FAB (Figure 1). The children were divided into 2 groups depending on whether they used a standard bilateral foot abduction brace or a unilateral above-the-knee brace. 5 children used a below-the-knee brace of different types, and 1 child did not use a brace at all due to mild clubfoot deformity.

### Compliance to bracing

Compliance to any brace was graded as excellent, good, fair, or non-compliant. If the brace was used until 4 years of age, or used 10 h or more every night at the time of follow-up examination, compliance was defined as excellent. If the brace was used until at least 2 years of age, or used between 6 and 10 h every night at the time of follow-up examination, compliance was defined as good. If the brace was terminated before 2 years of age, or used less than 6 h every night at the time of follow-up examination, compliance was defined as fair. If the brace was terminated before 1 year of age, the child was defined as being non-compliant.

### Size of hospital

The hospitals were divided into 2 groups. Hospitals in which more than 20 children were treated during these 3 years constituted one group (3 hospitals, 93 feet), and hospitals in which less than 20 children were treated made up the other group (5 hospitals, 69 feet).

### Evaluation

The mean age of the children at the time of the follow-up examination in March 2009 was 3 years and 10 months (range 2 years and 3 months to 5 years and 2 months). At follow-up, all feet were assessed according to Pirani’s scoring system (Staheli 2009), which has 6 variables (posterior crease, empty heal, equinus, reduction of the navicular bone, medial crease, and lateral curvature of the foot). Each variable is scored as 0 points, half a point, or 1 point, where 1 indicates maximum deformity. A fully corrected foot has a total score of 0 points, and a foot with maximum deformity has a total score of 6 points.

Using a hand-held goniometer, we measured passive dorsiflexion of the ankle joint, passive external rotation of the foot and ankle, and adduction/abduction position of the forefoot.

### Statistics

The Pearson chi-square test was used for comparison of categorical variables. To account for the bilateral observations, we analyzed the continuous data using a mixed model. In the analysis, a variance component was used to account for the repeated measures of the individuals. The analyses are presented with estimated marginal means. All analyses were performed according to the intention-to-treat principle. Values of p less than 0.05 were considered statistically significant. PASW statistics software version 18.0.1 was used for the statistical analysis.

The study was approved by the Regional Ethics Committee of western Norway (191.03).

## Results

### Overall results

**Figure 3.**
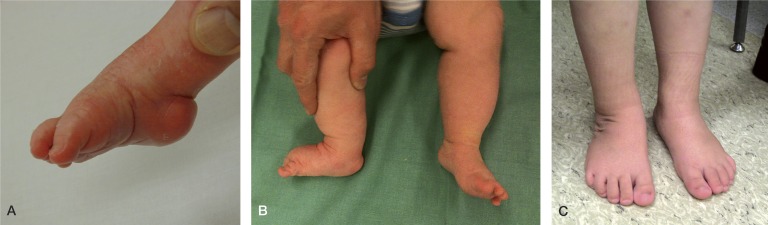
A. Clubfoot, right side, before treatment. B. Clubfoot, right side, after casting and Achilles tenotomy. C. Clubfoot, right side, at 3 years old.

The mean Pirani score before treatment was 4.8 (2.5–6) (116/162 feet). For all 162 feet, the mean number of casts during initial treatment, including the last cast after tenotomy, was 7.2 (3–13). 79% of the feet were treated with a percutaneous Achilles tenotomy. Figure 3 shows a unilateral clubfoot throughout the treatment period.

At the time of follow-up, 27 feet had been treated with either a second period of casting (15 feet), a second tenotomy (18 feet), or both (6 feet) due to relapse. 6 feet had been operated on with more extensive surgery than a tenotomy of the Achilles tendon. 3 feet were operated on with postero-medial release, 2 with posterior release, and 1 with transfer of the tibialis anterior tendon. Those treated with the unilateral above-the-knee brace had more casts and a higher frequency of Achilles tenotomy (Table 1).

**Table 1.** Overview of treatment for children treated with either bilateral brace or unilateral brace

	Bilateral brace	Unilateral brace
	(n = 102)	(n = 51)
*Initial treatment*		
Number of casts	6.7	8.1
Tenotomy of Achilles	74% (75/102)	94% (48/51)
*Treatment of relapse*		
Recasting	10% (10/102)	8% (5/51)
Second tenotomy of Achilles	9% (9/102)	18% (9/51)
Other operation	3% (3/102)	4% (2/51)

### Clinical outcome

At examination, 78% of the feet had a Pirani score of 0 or 0.5, 22% of the feet had a Pirani score of 1 or 1.5, and one foot had a score of 3.5. 92% of the feet had a passive dorsiflexion of 15 degrees or better, and one foot had an equinus position of 15 degrees (Table 2). The external rotation was 40 degrees or better in 93% of the feet, while none of the feet had a fixed internal rotated position. 84% of the feet had no adduction deformity. 4% of the feet had an adduction deformity of 10 degrees or more.

**Table 2.** Major clinical outcome variables and brace compliance in children treated with bilateral foot abduction brace and unilateral foot abduction brace

	Bilateral brace	Unilateral brace	
	(n = 102 feet)	(n = 51 feet)	p-value
Pirani score			
0 points	44% (45/102)	23% (12/51)	
0.5 point	33% (34/102)	51% (26/51)	
1 point	15% (15/102)	20% (10/51)	
> 1 point	8% (8/102)	6% (3/51)	
Mean points	0.4	0.6	0.1
Dorsal flection			
≥ 15 degrees	91% (93/102)	92% (47/51)	
0–14 degrees	9% (9/102)	6% (3/51)	
< 0 degrees	0%	2% (1/51)	
Mean degrees	24	24	0.8
External rotation			
≥ 40 degrees	92% (94/102)	94% (48/51)	
15–35 degrees	8% (8/102)	2% (1/51)	
< 15 degrees	0%	4% (2/51)	
Mean degrees	49	48	0.4
Adduction			
0 degrees	82% (84/102)	86% (44/51)	
5 degrees	15% (15/102)	8% (4/51)	
≥ 10 degrees	3% (3/102)	6% (3/51)	
Mean degrees	1	1	0.9
Compliance	n = 72 patients	n = 36 patients	
Exellent	56% (40/72)	81% (29/36)	
Good	26% (19/72)	11% (4/36)	
Fair	11% (8/72)	3% (1/36)	
Non-compliant	7% (5/72)	6% (2/36)	0.07

No difference was found between the two types of braces regarding dorsiflexion in the ankle, external rotation of the foot/ankle, and forefoot adduction. We found a tendency of better outcome measured by Pirani score in the group that used the bilateral foot abduction brace (p = 0.1). There was, however, a tendency of better compliance in the group using the unilateral brace (p = 0.07) (Table 2).

### Size of hospital

Children treated at the three largest hospitals had significantly better outcome as measured by Pirani score, and a tendency of better dorsiflexion in the ankle joint, compared to children who were treated at smaller hospitals. We found no statistically significant differences in external rotation of the foot/ankle and forefoot adduction between the two groups (Table 3).

**Table 3.** Major clinical outcome in children depending on size of hospital

	Hospitals where more than 20 children were treated (n = 93 feet)	Hospitals where less than 20 children were treated (n = 69 feet)	p-value
Pirani score			
0 points	48% (45/93)	23% (16/69)	
0.5 point	34% (32/93)	48% (33/69)	
1 point	11% (10/93)	22% (15/69)	
> 1 point	7% (6/93)	7% (5/69)	
Mean points	0.4	0.6	0.02
Dorsal flection			
≥ 15 degrees	95% (88/93)	89% (61/69)	
0–14 degrees	5% (5/93)	10% (7/69)	
< 0 degrees	0%	1% (1/69)	
Mean degrees	25	23	0.05
External rotation			
≥ 40 degrees	93% (86/93)	94% (65/69)	
15–35 degrees	6% (6/93)	4% (3/69)	
< 15 degrees	1% (1/93)	1% (1/69)	
Mean degrees	48	50	0.2
Adduction			
0 degrees	85% (79/93)	84% (58/69)	
5 degrees	11% (10/93)	13% (9/69)	
≥ 10 degrees	4% (4/93)	3% (2/69)	
Mean degrees	1	1	0.8

## Discussion

The overall initial results of clubfoot treatment presented in this multicenter study are good. We found similar outcome in the two types of braces, but there was a tendency to have better Pirani scores in children using the bilateral foot abduction brace. There was a tendency to have better compliance in children using the unilateral brace. We found better outcome as measured by Pirani score in children who were treated at the largest hospitals.

The number of patients in this study was fairly high, and few patients were lost to follow-up. This adds strength to the study. The relatively high Pirani score before treatment (mean 4.8) and the high number of casts (mean 7.2) indicate that this population with clubfoot was not especially mildly affected, but was most likely an average clubfoot population.

With successful closed treatment, later surgery is not necessary. Our results were good regarding avoidance of extensive surgery; only 5 feet needed posterior release or postero-medial release. At the follow-up examination, 1 additional patient was scheduled for extensive soft tissue surgery.

Ponseti claimed that his method of treating clubfoot was easy to learn (Ponseti 1997). Over the last 2 decades, numerous studies have shown good results with the Ponseti method. These studies have in common that they have been at large centers, treating many children. Our study was performed at 8 hospitals. Even though the overall results were good, we found better outcome as measured by Pirani score in children who were treated at the largest hospitals. This indicates that a certain number of procedures are needed to achieve sufficient experience. Furthermore, and as a consequence, the number treated at each hospital may not be as important as the number treated by each surgeon.

Most of the feet in this study were initially treated with a semi-rigid fibreglass cast. Whether or not one casting material is superior to the other is controversial, but the results of a recent study have supported the use of plaster of Paris with the Ponseti technique (Pittner et al. 2008).

An abduction brace is considered mandatory to prevent relapses, and is a crucial part of the Ponseti treatment. Non-compliant patients tend to relapse (Dobbs et al. 2004, Morcuende et al. 2004). Failure of appropriate bracing is the most common cause of relapse (Staheli 2009). Short-term studies have also shown poor compliance with the foot abduction brace and therefore a high relapse rate (Haft et al. 2007).

The use of a bilateral foot abduction brace at night until 4 years of age is now recommended (Ponseti 1996, Staheli 2009). Even though the relationship between compliance and relapse is well documented, the degree of compliance is not differentiated in the literature. Some authors do not give any definition of a non-compliant patient. Dobbs et al. (2004) reported non-compliance as complete discontinuation of the use of the orthosis. It is our experience that many children partly comply with the brace. Some might use it as recommended for some years, but not until 4 years of age. Others might use the brace until 4 years of age, but not for as many hours as prescribed. We made our own grading of compliance that was suitable for this group of patients, where some of the children had not reached the age of 4 at the time of follow-up. We consider that the compliance in our material was good; by our definition only 7% were non-compliant. It takes a lot of effort to make the children and parents comply with the brace. 45% of the children who used the bilateral foot abduction brace needed at least one change in the type of brace. The unilateral foot abduction brace might be easier to comply with, especially in the unilateral cases. We found slightly better compliance in the children using the unilateral brace. Furthermore, as many as 12% of the children who were fitted with the bilateral foot abduction brace ended up with the unilateral brace. Ponseti warned about using a unilateral short leg brace, as it fails to hold the foot in abduction (Staheli 2009). The brace referred to in the present report was above-the-knee, and was able to hold the foot in abduction/ external rotation. The unilateral brace used in this study was custom-made, and therefore quite expensive and time-consuming to manufacture. The use of different types of braces at the different participating hospitals was a potential problem in this study. Even so, the Ponseti method is a standardized method, and the unilateral above-the-knee brace is also fairly standard. Moreover, the treatment of clubfoot in this study reflects a real-life setting, and the results may thus be more applicable to ordinary surgeons at ordinary hospitals than the results of particularly dedicated high-volume centers, which are the setting of many studies.

There are several systems designed to assess or evaluate a clubfoot (Harrold and Walker 1983, Dimeglio et al. 1995, Lehman et al. 2003). 116 of 162 feet were assessed with Pirani score before treatment started, and all feet in this study were assessed according to Pirani at follow-up. Like the other systems, Pirani’s scoring system has mainly been used as a tool during the initial treatment. On the basis of our study results and experience, we believe that it can be used also for evaluation of feet in children less than 5 years of age.

The Ponseti method is associated with a relatively high rate of relapse, as also reported by the inventor himself (Laaveg and Ponseti 1980, Changulani et al. 2006). Still, the relapses have proven to be less rigid and easier to treat. According to Ponseti, a relapse should be treated with a second period of casting, with or without percutaneous tenotomy or open Achilles tendon lengthening. After the age of 30 months, one might consider a transfer of the tibialis anterior tendon (Ponseti 1997). The rate of a second tenotomy was fairly high in our material—higher than recasting alone. This might indicate that we were not sufficiently familiar with the Ponseti casting technique and that we did not believe that we could fully correct a relapse with casting alone. Only 1 foot was operated with a transfer of the tibialis anterior tendon. Other authors have reported a higher frequency of this operation (Cooper and Dietz 1995). It is possible that a few more of our patients would have benefitted from this operation.

A longer follow-up period is needed for our patients. To decide whether the unilateral brace is equivalent or inferior to the bilateral foot abduction brace that is used worldwide, a randomized controlled study will be required.

In conclusion, the results of this multicenter study were good and not inferior to those of other studies, both with regard to avoiding extensive soft tissue surgery and as measured from clinical outcome of the foot. The introduction of the Ponseti method at hospitals participating in this study has been successful.
